# Host–Pathogen Interactions between *Metarhizium* spp. and Locusts

**DOI:** 10.3390/jof8060602

**Published:** 2022-06-03

**Authors:** Jun Li, Yuxian Xia

**Affiliations:** 1 Genetic Engineering Research Center, School of Life Sciences, Chongqing University, Chongqing 401331, China; jun.lee@cqu.edu.cn; 2 Chongqing Engineering Research Center for Fungal Insecticide, Chongqing 401331, China; 3 Key Laboratory of Gene Function and Regulation Technologies Under Chongqing Municipal Education Commission, Chongqing 401331, China

**Keywords:** *Metarhizium*, locust, pathogenicity, immune response

## Abstract

The progress in research on the interactions between *Metarhizium* spp. and locusts has improved our understanding of the interactions between fungal infection and host immunity. A general network of immune responses has been constructed, and the pathways regulating fungal pathogenicity have also been explored in depth. However, there have been no systematic surveys of interaction between *Metarhizium* spp. and locusts. The pathogenesis of *Metarhizium* comprises conidial attachment, germination, appressorial formation, and colonization in the body cavity of the host locusts. Meanwhile, the locust resists fungal infection through humoral and cellular immunity. Here, we summarize the crucial pathways that regulate the pathogenesis of *Metarhizium* and host immune defense. Conidial hydrophobicity is mainly affected by the contents of hydrophobins and chitin. Appressorial formation is regulated by the pathways of MAPKs, cAMP/PKA, and Ca^2+^/calmodulin. Lipid droplets degradation and secreted enzymes contributed to fungal penetration. The humoral response of locust is coordinated by the Toll pathway and the ecdysone. The regulatory mechanism of hemocyte differentiation and migration is elusive. In addition, behavioral fever and density-dependent population immunity have an impact on the resistance of hosts against fungal infection. This review depicts a prospect to help us understand host–pathogen interactions and provides a foundation for the engineering of entomopathogenic fungi and the discovery of insecticidal targets to control insect pests.

## 1. Introduction

Fungi are the main pathogens that balance populations of insects in nature. To date, there are more than 1000 species of fungi that are distributed in the phyla Entomophthoromycota, Blastocladiomycota, Microsporidia, Basidiomycota, and Ascomycota [1,2]. The entomopathogenic fungus *Metarhizium* (ascomycete) can infect a wide spectrum of host insects (generalists), whereas others have a narrow host range (specialists). Generalist species, including *M. anisopliae*, *M. robertsii*, and *M. brunneum*, can infect a variety of insects, including locusts. The specialist species *M. acridum*, formerly known as *M. flavoviride* and *M. anisopliae* var. *acridum*, only infects locusts [3,4]. It is commonly believed that the specialist is the ancestor of generalist species that evolved through horizontal gene transfer [5,6]. A number of *M. anisopliae* strains have been registered as biopesticides for the control of locusts and many other pest insects worldwide [7,8]. The specialist species *M. acridum* has also been applied in locust control [9]. Generalist and specialist species show similar pathogenic processes in locusts, and the interaction between locusts and *Metarhizium* spp., mainly *M. acridum, M. anisopliae* and *M. robertsii,* has been well studied.

The locusts including *Locusta migratoria* and *Schistocerca gregaria* cause tremendous damage to major crops [10,11]. Locusts are the only grasshopper species in Acrididae that display density-dependent phase changes [12]. The aggregation of solitarious locusts could transform these locusts into the gregarious locusts. The gregarious locusts with increased density of populations markedly improve the immune defense of locusts against pathogenic fungi [13]. In addition, locusts employ behavioral fever to fight fungal infections [14].

**Figure 1:**
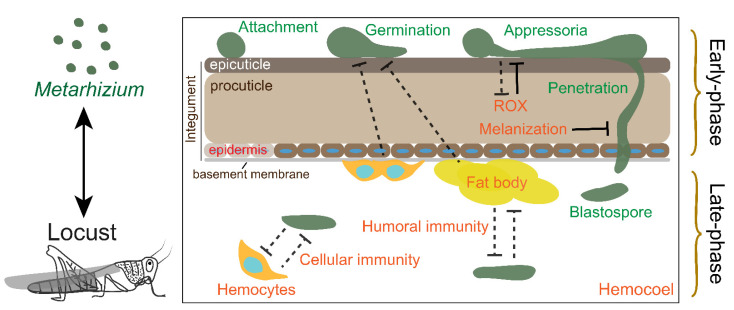
Schematic overview of the interactions between *Metarhizium* spp. and locusts. The dotted T represents indirect opposition. The solid T represents direct interactions.

As a standard model in the research of host insect and fungal pathogen interactions, many papers have been published on the interactions between *Metarhizium* and locusts. There are two stages, parasitism and saprophytism, after fungal infection. Due to occupied body cavity of locusts, resistance to *Metarhizium* is lost during the saprophytism. In this review, we only outline the advances in the interaction between *Metarhizium* spp. and locusts during parasitism before host death. We divide the process of host–pathogen interaction into two main phases during fungal infection, the “early phase” and “late-phase” (Figure 1). The first main phase, named the “early phase”, is a fungal breach in the integument but not in the hemocoel. During this phase, the host protects against fungal development through the prophenoloxidase (PPO) system and the antimicrobial compounds in the cuticle, and the preparation of the immune response in the hemocoel, which is mediated by hemocytes and fat body (functionally equivalent to the mammalian liver). The second main phase, or “late-phase”, involves fungal colonization in the hemocoel and the humoral and cellular immune response of the host. In addition, locusts induce behavioral fever to reduce mycosis and utilize density-dependent prophylaxis to enhance the protection of the population against fungal infections. We provide an overview of the mechanism of interaction between *Metarhizium* and locusts to facilitate a thorough understanding of the interactions between host insects and fungal pathogens.

## 2. The Early Phase Interaction

**Figure 2:**
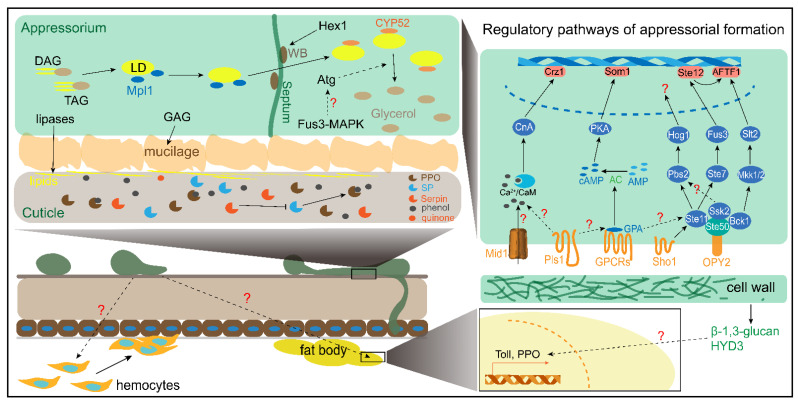
A schematic model of the early phase interaction between *Metarhizium* spp. and locusts. The regulatory mechanisms of appressorial formation and the early phase immune response are depicted. The regulatory pathway of appressorial formation only represents the main signaling pathway, including Ca^2+^/CaM, cAMP/PKA, and MAPKs. The processes of LD synthesis and degradation are also shown in the model. The dotted arrows represent indirect actions to promote another component. The solid arrows or T indicate direct promotion or restraint to another component. DAG, diacylglycerol; TAG, triacylglycerol; LD, lipid droplet; GAG, galactosaminogalactan and the biosynthesis genes cluster; WB, woronin body; SP, serine protease; PPO, prophenoloxidase.

There are four stages, including conidial attachment, germination, appressorial formation, and penetration, in the early phase of *Metarzihium* infection. The attached conidia utilize epicuticle lipids as a unique carbon source to germinate and differentiate appressoria. Furthermore, the penetration peg bud from appressorial turgor would breach the integument of the locusts. There are a number of reports on conidial attachment, appressorial formation, and penetration; therefore, we mainly summarize these processes (Figure 2).

### 2.1. Attachment

Conidial attachment onto the surface of the host is the first step for fungal invasion. The attachment primarily relies on the hydrophobicity of the conidial surface, which interacts with the surface of the host epicuticle [15]. The conidial surface is coated by hydrophobins that are self-assembled as layer rodlets and provide hydrophobicity [16,17]. Hydrophobin genes (*Hyd*) are highly expressed during conidiation, and the regulatory mechanism is different in various *Metarhizium* species. A regulator of the G-protein signal, *cag8*, facilitates the expression of hydrophobin *ssgA* in *M. anisopliae* and hyd3 in *M. brunneum* [18,19]. In *M. acridum*, *MaHyd3*, *MaHyd4* and *MaHyd5* are downregulated by the deletion of *MaCwh1* and *MaCwh43*, which are two calcofluor white hypersensitive proteins [20]. The expression of the two *MaHyds* genes was significantly reduced in a *MaCrz1* mutant [21]. Furthermore, *MaCwh1* and *MaCwh43* expression are decreased upon the disruption of *MaCrz1* [20]. These results suggest that *MaCrz1* might partially regulate *MaHyd* expression through *MaCwhs*. Some *Hyds* are also expressed in early phase infection and are regulated by several genes that are different from those in conidiation. In *M. anisopliae*, *Hyds* were highly expressed at pH 6 to pH 8 but not at pH 3, which was consistent with the pH of the infected cuticle [22]. In *M. brunneum, hyd3* expression increases in the infection stage and contributes to conidial hydrophobicity [19]. The expression of *MrHyd4* is regulated by the transcription factor *MrCre1*, which is mediated by the histone lysine methyltransferase *MrKMT2* during the germination and appressorial formation of *Metarhizium robertsii* on the surface of the host. [23]. In addition to hydrophobins and regulatory genes, conidial hydrophobicity is likewise altered by several genes through altering the chitin content in the cell wall. The chitin synthase *MaChsIII*, *MaChsV* and *MaChsVII* mutants reduce conidial hydrophobicity [24]. In addition, the chitin content and rodlets are significantly reduced in the knockout of *MaCnA*, *MapacC* and *MripacC* [25,26,27].

During early infection, the adhesin-like protein Mad1 also promotes adhesion of conidia and germlings to the surface of the host. The adherence of conidia to wings is dramatically decreased by the disruption of Mad1 [28]. The expression of *Mad1* is increased in the early stages of infection in insects [15,28]. The expression of *Mad1* is downregulated by a number of genes, including transcription regulator *MaAreB* and *MaNmrA*, Fus3/Kss1-type mitogen-activated protein kinase (MAPK) *MaMk1*, the exopolysaccharide galactosaminogalactan (GAG) biosynthetic gene cluster *MrGAG*, transcription factor *MaSom1*, which is located downstream of the cycle adenosine monophosphate (cAMP) -dependent protein kinase A (PKA) pathway [29,30,31,32,33]. Moreover, *Mad1* expression is increased by the disruption of *MrGprk*, which is a class VI fungal G-protein-coupled receptor K (GPRK) [34].

The surface of the cell wall is coated by hydrophobins, which might be attached to polysaccharide through melanin [35]. However, how those genes regulate *hyd* expression is unknown through an unexplained network. In addition, the conidial hydrophobicity is also affected by the changed components of the cell wall. The rodlets of the conidial surface are regulated by Ca^2+^/calmodulin (CaM)-dependent pathway and chitin synthase might be involved in. The pathway likely influences HYDs binding to the layer of polysaccharide or the transportation of HYDs from the cell plasma to the cell wall. In sum, this mechanism of hydrophobins expression and rodlets construction needs further exploration.

### 2.2. Appressorial Formation

Appressorium is the primary infection structure for the breach of *Metarhizium* from the cuticle into the body cavity of the locust. Conidial germination occurs on the poorly nutritious and hydrophobic surface of the host insect to form an appressorium. The conidial germination and appressorial formation are promoted by polar lipids, including long-chain fatty acids, methylated alkaneas, methyl-ethyl esters and short-chain alkanes, which are extracted from the hind wings of locusts using dichloromethane (DCM) or methanol [36,37]. However, the nonpolar lipids extracted from locust hindwings using hexane also cannot promote appressorial differentiation [37]. This suggests that the appressorial differentiation is stimulated by polar lipids. Furthermore, saturated long-chain fatty acids, including C 16:0, C 18:0, and C 20:0, facilitate the appressorial formation [38]. However, little is known about how saturated long-chain fatty acids promote appressorial formation.

Each of the fungal MAPK pathways consists of a cascade mediated by MAPK kinase kinase (MAPKKK), MAPK kinase (MAPKK), MAPK. MAPKKK is activated by the binding of an activator protein [39]. MAPK plays a critical role in signaling transduction and acts as a bridge to connect extracellular signals and downstream transcription factors. Exogenous MAPKK inhibitor PA-98059 eliminated the appressorial formation induced by the DCM extracts [37]. This finding indicates that MAPK is required to trigger the differentiation of germlings to the appressorium. The appressorial formation is eliminated by the deficiency of *MaMk1* or *Pmk1* of *M. rileyi*, which are FUS3/KSS1-type MAPKs [32,40]. The appressorial formation on locust hindwings is not produced by the mutant of *MrSte11* (MAPKKK), *MrSte7* (MAPKK), and *MrFus3* (MAPK) [41]. STE11, STE7 and FUS3 have been identified as MAPK modules that respond to extracellular pheromones in yeast [39]. MrSTE11 directly interacts with and is activated by MrSTE50, which is an adaptor protein that interacts with the membrane protein MrOPY2. The deficiency of *MrSte50* and *MrOpy2* strains do not produce appressoria [42]. Furthermore, the complete or nearly complete loss ability of differentiation to appressoria is caused by the deletion of the transcription factor Ste12 in *M. acridum*, *M. rileyi*, and *M. robertsii* [43,44,45]. MrSte12 positively regulates the expression of the transcription factor *MrAFTF1* through MrFus3 [45]. Interestingly, both the knockout and overexpression of MrAFTF1 strains decreased appressorial formation. These results revealed that a Fus3-type MAPK module comprising OPY2, STE50, STE11, STE7, FUS3, and STE12 is indispensable for the appressorial formation of *Metarhizium* (Figure 2). In addition to the Fus3-type MAPK pathway, two other MAPK cascade pathways, including Bck1-Mkk1/2-Slt2 and Ste11-Pbs2-Hog1, also contribute to appressorial formation (Figure 2). MrBck1 and MrSsk2 also directly interact with and are activated by MrSTE50 [42]. The deletion of *MrBck1* (MAPKKK) and *MrMkk1/2* (MAPKK) decreased appressorial formation [41]. The *MrSlt2* (MAPK) mutant lost the ability to form appressoria and regulates the *MrAFTF1* expression independent of MrSte12 [41]. In addition, the deletion strains of *MrPbs2* (MAPKK), *MrHog1* (MAPK), and MrSsk2 (MAPKKK) decreased the appressorial formation [41]. The Hog1-MAPK cascade is activated by Sho1 in response to extracellular signals, such as high osmotic pressure. The disruption of *MaSho1* but not the SH3 domain reduced the appressorial formation, and the knockdown of *ManSho1* in *M. anisopliae* caused aberrant appressoria [46,47].

In addition to OPY2, two G-protein-coupled receptors (GPCRs), including Pth11-like MrGpr8 and MrGprk, with an RGS domain, promote appressorial formation through a connection with the MAPK pathway (Figure 2). The disruption of *MrGpr8* eliminates the differentiation of germlings to appressoria by impairing the nuclear translocation of MrFUS3 and reducing endogenous cAMP levels [48]. The *MrGprk* decreases appressorial formation and intracellular cAMP concentration [34]. Mutation of the G-protein subunit *MrGPA1* mutant dramatically reduced the appressorial formation and intracellular cAMP [49]. GPCRs not only trigger MAPK but also regulate cAMP and might activate the PKA pathway to promote appressorial formation. However, the appressorial formation of *M. acridum* has not been affected by the addition of exogenous cAMP [37]. In addition, the knockdown of the AMP cyclase *MaAC* significantly decreased endogenous cAMP but had no influence on the appressorial formation [50]. Nonetheless, the endogenous cAMP in *M. anisopliae* gradually increass during appressorial differentiation and rapidly decreases after the termination of appressorial formation [51]. This hints that cAMP might function as a signal downstream of the MAKP pathway, but it is not necessary for direct activation of the MAPK pathway and cannot promote appressorial formation. Interestingly, exogenous addition of the PKA inhibitor H89 fully inhibited the differentiation of germlings to appressoria that is induced by lipids [37]. The impaired function of PKA1, which is a class I PKA subunit of *M. anisopliae*, delayed the appressorial formation on plastic coverslip [52]. Furthermore, the disruption of *MaSom1*, which is a transcription factor in the cAMP/PKA pathway, significantly decreased appressorium formation [31]. These findings indicate that the PKA pathway, but not cAMP, participates in central signaling-associated appressorial formation.

In addition to the MAPK and PKA pathways, the Ca^2+^/CaM-dependent pathway has an impact on appressorial formation (Figure 2). Deletion of *MaMid1*, which is a channel protein that modulates Ca^2+^ influxes, decreases the intracellular Ca^2+^ levels and reduces the appressorial formation [24]. The Knockout of calcineurin *MaCnA* and the transcription factor *MaCrz1* also reduces the appressorial formation to decrease virulence [21,53].

Other pathways and metabolism-associated genes also delay, reduce, eliminate, or increase appressorial formation. Appressorial differentiation was delayed by these impaired genes, including the tetraspanin *MaPls1*, the pH-responsive transcription factor *MapacC*, and the O-mannosyltransferases *MaPmt2* and *MaPmt4* [26,54,55,56]. The formation rate of appressorium is reduced by some genes mutants including the negative transcription regulators *MaNmrA*, *MaAreB*, and *β-tubulin* of *M. acridum*, the actin-regulating kinase *MrArk*, the sucrose non-fermenting protein kinase MaSnf1, the bifunctional catalase and peroxidase *MaKatG1*, *MrHex1*, which is related to the formation of the woronin body, and *MaPEX33*, which is associated with the peroxisomal import pathway, the polyketide synthase *MrPks2* [29,30,40,45,57,58,59,60,61]. The appressorial formation ability is lost when the transcription factor *MrSkn7* is mutated and the inhibition of Isocitrate lyase *ManICL* is inhibited [62,63]. The disruption of *MaAfr* (adenylate-forming reductase) can significantly increase the appressorial formation rate [64].

Several important regulatory pathways in appressorial formation are shown in Figure 2. The exogenous stimulation might be facilitating appressorial formation. The membrane receptors, such as Opy2, Sho1, Pls1, GPCRs, and Mid1, participate in promoting differentiation of germlings to appressorium. However, the signals, including chemicals and physicals, are still not discovered. The receptor proteins might act as scaffolds to support intracellular transduction signals, such as Ste50 and GPA1. In contrast, the appressorial formation is promoted by the saturated long-chain fatty acids which might function as precursors for synthesis of LD. This hints that lipid metabolites functioned as endogenous signals that plays a crucial role in appressorial formation. The long-chain saturated fatty acids that are transported intracellularly can synthesize diacylglycerols (DAGs) and triacylglycerols (TAGs) to accumulate in lipid droplets (LDs). DAG is a secondary messenger that targets protein kinase C (PKC) and consequently changes the Ca^2+^ concentration in vivo to trigger other pathways. In addition, the previous hypothesis that is an unknown signal destructs Ca^2+^ gradients in vivo, which maintains topical growth of hyphae, to cause abnormal differentiation of germlings [65]. The exogenous addition of saturated DAGs enhances the appressorium formation in *Magnaporthe oryzae* [66]. Fus3-MAPK might be regulated by DAG or other metabolites to facilitate appressorial turgor through disturbing polarized hyphal growth. Early evidence indicates that Fus3-MAPK might promote LD degradation. However, the correlation between Fus3-MAPK and lipid metabolism needs to be further explored. In addition, the most important is which signal initiates Fus3-MAPK to induce the differentiation of germlings to the appressorium and how Fus3-MAPK regulates those genes working in abnormal polar growth of germlings. Furthermore, the connection of the MAPK, PKA, and CaM-dependent pathways needs to be thoroughly researched.

### 2.3. Penetration

The appressoria builds up proper turgor pressure to allow the vertical growth of penetration pegs and the breach of the host cuticle. Additionally, secreted hydrolases, such as proteases and chitinases, facilitate penetration by hydrolyzing chitin and the protein of the cuticle [67,68]. The turgor pressure is mainly provided by the production of solutes such as glycerol from LD lipolyzed by lipases [69,70]. The glycerol-3-phosphate acyltransferase *MrGAT* promotes the synthesis of triacylglycerol (TAG) to increase the LD content in vivo [71]. The LD accumulation and transport to the vacuoles are mediated by the binding of the LD-specific perilipin-like protein Mpl1 to the LD surface. Mpl1 is significantly downregulated in the *MrArk1*, *MaPmt1*, *MrGprk*, *MrMk1*, *MaAreB*, and *MrKmt2* mutants [23,29,32,34,72,73]. However, the *MaAfr^IV^* significantly increased Mpl1 expression [64]. Reduced expression of *MrHex1* dramatically decreases turgor pressure [60]. The transportation of LDs into vacuoles of appressorial turgor is degraded through autophagic bodies and is mediated by MrAtg3-5, MrAtg7, MrAtg8, MrAtg12, MrAtg15 and MrAtg16 [69,74,75]. This process is directly mediated by intracellular lipases such as MrMEST1 [6]. The degradation of LDs is expedited by Mras1 and Mras3-7, which are transcriptionally regulated by *MrSt12* [75]. The *Mrass*, except for *Mras1* and *Mras2*, was significantly downregulated in the *MrGpr8* mutant [48]. The regulatory model of LD synthesis and degradation is shown in Figure 2.

During penetration, the host cuticle matrix is hydrolyzed by secreted proteases and chitinases, including subtilisin-like proteases (Pr1), trypsin-like proteases (Pr2 and Try1), chymotrypsin, metalloproteases, aspartyl proteases, aminopeptidases, endochitinases (Chit3), and chitinases (ChitI and ChitII) [22,76]. *Pr1* and *Pr2* in *Metarhizium* spp. are induced by the locust cuticle; purified Pr1 can hydrolyze the cuticle proteins of locust wings and abdomen [36,77]. Chitinases are involved in the digestion of the host cuticle chitin [78]. The expression of *Pr1s*, *Pr2*, Try1 and Chits is regulated by some genes that regulate *Hyd* and *Mad1* expression and appressorial formation. *Pr1A, Pr1B, Pr1F*, *Try* and *Chit30* are downregulated in a *MrGprk* mutant [33]. *Pr1A* and *Pr1C* were significantly decreased in a *MrGpa1* mutant [49]. *Pr1A*, *Try*, and *Chit30* were significantly downregulated in a *MrArk1*-deficient strain [72]. *Pr1A* and *Chit* were decreased by the deficiency of *MaSnf1* [58]. *Chit3* and *subtilisin* were decreased upon the disruption of *Macwh1* and *Macwh43* [60]. *Pr1*, *Pr2*, *ChitI* and *ChitII* were downregulated by one- to three-fold in a *MaMid1* mutant [24]. *Pr1C*, *Pr1E*, *Pr1F*, *Pr1I*, *Pr1K*, *chitinase* and *chymotrypsin* were downregulated in a *MaPKA1* mutant [52]. The expression of *Pr1C* and *Try* in *MaSom1* deficiency was significantly lower than that in WT [31]. The expression of *Pr1* and *Chit1* is reduced in cells deficient in *MaAreB*, *MaNmrA*, *Macwh1*, *Macwh43*, *MapacC*, and *MaCrz1* [21,26,29,30,60,79]. In contrast, certain genes reduce the expression of those proteases. The expression of *Pr1* was increased in *MaPmt4* and *MaAfr^IV^* [55,64]. *Pr1D* was upregulated in a *MaPKA1* mutant [52]. The expression of *Pr1B* and *Pr1C* was increased in an impaired *MaCrz1* [21]. The partial *Pr1*s and *Pr2*s are regulated by *MrFus3* and *MrSlt12* [41]. In addition, the appressorial mucilaginous, which is synthesized by *MrGAG*, assists in the secretion and proper function of degradation enzymes, such as serine proteases and chitinases [80].

MrGpr8 and MrSte12 both regulate the expression of *Mras*, which negatively regulates MrMpl1 to facilitate the degradation of LDs. This finding reveals that the Fus3-MAPK pathway might be involved in the microlipophagy of the appressorium and the degradation of LDs by regulating autophagy-associated genes. However, direct evidence does not indicate the interaction of Fus3 with Atgs, so this hypothesis requires further exploration. Furthermore, the signal triggered by the degradation of LDs remains elusive. In addition, the osmotic pressure created by glycerol initiates the growth of the penetration peg. However, what kinds of osmotic sense proteins trigger signals and how penetration pegs accurately pierce into the cuticle is still elusive in *Metarhizium*. In addition, the tremendous proteases expressed in the penetration stage are modulated by several genes, but the model of interaction between these genes is not fully understood.

### 2.4. The Locust Early Phase Immunity

The insect integument is the first obstacle against fungal invasion. The integument comprises the epicuticle, procuticle, epidermis, and basement membrane (Figure 1) [81]. The phenoloxidases (POs) located in the cuticle mainly participate in cuticular sclerotization through the transformation of polyphenol and quinone derivatives to melanin [82]. In addition, PO also directly attaches to and melanizes the fungal cell wall to impede hyphal growth [83]. PO is derived from proPO (PPO), which is cleaved by serine protease (SP). SP is inhibited by an SP inhibitor (serpin) [84]. The active forms of SP and PO were both identified in the locust cuticle [85]. In addition, *serpin1* is also highly expressed in the locust epidermis [86]. Therefore, the complete PPO system in the cuticle might be considered a defense against fungal invasion.

The fat body and hemocytes of locusts exhibit early phase immunity to fungal attachment, germination, and penetration into the integument of locusts. A number of differentially expressed genes (DEGs), including the increased expression of scavenger receptor A and PPO and the decreased expression of C-type lectin and MyD88, are in fat bodies and hemocytes once conidial attachment occurs on the surface of locusts [87]. In addition, the increased expression of Toll pathway genes, including the *Spatzle, Toll9, MyD88, Cactus* and *GNBP*-like genes, was observed in fat bodies during *M. acridum* germination on the surface of locusts [88]. Furthermore, β-1,3-glucan is a pathogen-associated molecular pattern (PAMP) of the fungal cell wall that is recognized by β-1,3-glucan recognition protein (βGRP, also called GNBP3), and it is found on the surface of hemocytes during the conidial attachment [88]. In addition, the expression of immune genes, including *Spatzle*, *MyD88* and *PPO11*, was enhanced by dropping a specific hydrophobin MaHYD3 of *M. acridum* on the cuticle [89].

In addition to their contribution to the humoral immune response, hemocytes also respond to fungal early phase invasion. The circulating hemocytes were significantly reduced in the hemocoel when MaHYD3 was dropped on the surface of the locust [89]. Moreover, the phagocyte number was reduced after inoculation with *M. acridum* to promote the differentiation of the appressorium [90]. Furthermore, the circulating hemocytes began to adhere to the basement membrane and even attached to the epidermis when *M. anisopliae* adhered to the epicuticle of desert locusts [91]. Moreover, the phagocytosis of circulating hemocytes was increased after treatment with MacHYD3 [89].

Early reports on the procuticle and epithelium in infected silkworms and fruit flies showed increased expression of AMP-like *cecropin*, which is regulated by the IMD pathway [92,93]. This suggests that the integument is not only a statically mechanic barrier but also an immune organ that can impede fungal invasion [83]. In early phase immunity, hemocytes migration and alterations in transcription processes in the fat body indicate that the integument is closely connected to the fat body and hemocytes through the transfer of fungal signals in the hemocoel. However, little is known about how locusts sense fungal PAMPs, how they increase the expression of immune-related genes in the fat bodies and hemocytes, and how they induce hemocytes migration to the basement of the integument.

## 3. The Late-Phase Interaction

### 3.1. Colonization of Metarhizium in Hemocoel

Fungal hyphae that have penetrated the integument are converted to yeast-like hyphal bodies (HBs) to adapt to the host hemocoel. Additionally, the fungal cell wall remodels to evade host immunization. Furthermore, secreted enzymes and metabolites of HBs inhibit host immunity or take in host nutrients. These processes facilitate the successful colonization of fungi in the hemocoel.

The MAPK pathway is involved in the conversion of cellular morphology. The knockdown of *ManSho1* gives rise to the failure of the conversion of hyphae to HB [47]. The fungal virulence under direct injection into hemocoel is declined by the disruption of Hog1-MAPK and Slt2-MAPK pathway but not Fus3-MAPK pathway [41]. Compared to wild type strain, the numbers of DEGs detected in the *MrHog1*, *MrFus3* and *MrSlt2* knockout strains [41]. Furthermore, there were more DEGs in the *MrHog1* mutant than in the *MrFus3* and *MrSlt2* mutants. The disruption of *MrHog1* decreases the expression of these pathways component, including PKA subunit, CaM-dependent kinase, PKC, and histidine kinase, in hemocoel colonization [41]. These findings indicate that the Hog1-MAPK pathway plays a critical role in the fungal colonization. In addition, several genes associated with the cell wall are regulated by the MAPK pathway. Two glucanase genes upregulated in hemolymph and were regulated by *MrSlt2* [41]. The collagen-like protein Mcl1 coats on the surface of HBs to evade the immune response of the host [94]. *Mcl1* is regulated by *MrSlt2* and *MrGprk* [34,41]. Mad1 also contributes to conversion of mycelium to HB in hemolymph [28]. In addition, the sterol carrier protein MrNPC2a increases the sterol content of the cell membrane to enhance resistance against the host immune response [95].

**Figure 3:**
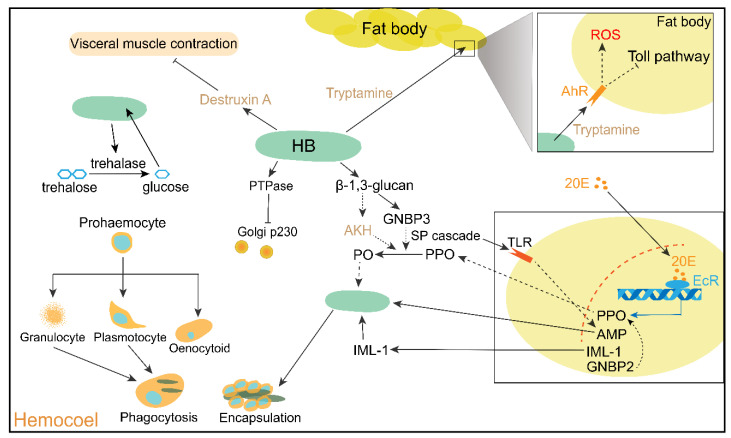
The late-phase interactions between *Metarhizium* spp. and locusts. The growth of the yeast-like hyphal bodies (HB) in the hemolymph of locusts relies on the ingestion of the locusts’ nutrients. The secreted protein and metabolites from *Metarhizium* to restrain humoral and cellular immunity. The pathways of ecdysone and Toll are opposed to the growth of HB. The dotted arrows or T represent indirect actions to promote or inhibit another component. The solid arrows or T indicate direct promotion or restraint to another component. 20E, 20-Hydroxyecdysone; EcR, ecdysone receptor; TLR, Toll-like receptor; IML-1, immunlectin-1; GNBP3, Gram-negative bacteria binding protein 3; AhR, aryl hydrocarbon receptor; SP, serine protease; Serpin, serine protease inhibitor; ROS, reactive oxygen species; PPO, prophenoloxidase; PO, phenoloxidase; AMP, antimicrobial peptides; PTPase, protein tyrosine phosphatase.

A number of the secreted enzymes of hyphal bodies in the hemolymph inhibit immune-related proteins and hydrolyze disaccharides as a nutritious source (Figure 3). The acid and neutral trehalases of *M. acridum* hydrolyze the hemolymph trehalose of locusts as a carbon source [96,97]. The overexpression of acid trehalase promoted *Metarhizium* growth in the hemolymph of locusts [98]. An acid tyrosine phosphatase PTPase of *M. acridum* suppressed the *trans*-Golgi protein p230, which is involved in phagophore formation and vesicular transport [99,100,101,102].

The metabolites released from hyphal bodies are targeted to muscle or immune tissues, such as fat bodies (Figure 3). The tryptamine synthesized by the specialist species *M. acridum* targets the aryl hydrocarbon receptor AhR of fat bodies in locusts to produce reactive oxygen species (ROS) and suppress immune response genes, including *cactus*, *stubble*, and *easter* [103]. Mycotoxin destruxin A, which is produced by generalist species such as *M. anisopliae* but not specialist species such as *M. acridum*, induces visceral muscle contraction in locusts through its effect on the influx of extracellular Ca^2+^ [104].

Cell wall remodeling of the hyphal bodies is regulated by the Hog1-MAPK pathway. It interacts with these pathways, including PKA, PKC, Ca^2+^/CaM, and histone kinase, which are upregulated during early phase infection [41]. Som1, Crz1 and CnA regulate the chitin and β-1,3-glucan contents in the cell wall of the conidia or blastospores. These findings indicate that Hog1-MAPK regulates the reconstruction of the cell wall through PKA- and Ca^2+^/CaM-dependent pathways. However, this hypothesis still needs to be confirmed. In addition, more proteins that are secreted into the hemocoel by yeast-like hyphal bodies interacting with the immune system of locust have not been fully identified by validated evidence. There are dozens of biosynthesis gene clusters (BGCs) that have been predicted in the genome of *Metarhizium*; however, a few metabolites have been reported to be toxic to locusts [105,106]. These findings reveal that there are more unrecognized compounds that might be toxic to insects that remain to be identified.

### 3.2. Humoral Response at Late-Phase Immunity

The humoral response, including antimicrobial peptides (AMPs), is mainly produced by fat bodies, and these factors are released to the hemolymph to destroy pathogens [107]. Several AMPs have been identified in locusts, with a broad spectrum toward Gram-positive, Gram-negative and fungi [108]. In addition, AMP *defensin 5 (DEF5)* expression is increased in the fat body of locusts infected by *Metarhizium* [109]. This indicates that defensin of locusts is likely to defend against fungal infection.

Antifungal AMP expression is mostly regulated by the Toll pathway and ecdysone/EcR pathway in locusts (Figure 3). Fungal PAMPs, such as β-1,3-glucan, are recognized by GNBP3 and C-type lectin in locusts [110,111]. Consequently, GNBP3 triggers the Toll pathway through the SP cascade [107]. Ecdysone/EcR, which are mainly involved in insect molting, also participate in the immune response by directly regulating AMP expression [112]. 20-Hydroxyecdysone (20E) binds to its nuclear receptor, ecdysone receptor (EcR), to exert its effect on gene transcription [113]. Knockdown of EcR significantly reduced locust mortality under *M. anisopliae* infection [112]. Moreover, 20E increased the expression of *defensin* and *diptericin* by enhancing *PGRP-SA* expression [112]. In addition to the Toll and ecdysone/EcR pathways, two other genes are also involved in the regulation of defensin. Inhibitor of apoptosis protein 1 (IAP1) promotes *defensin* expression, and knockdown of IAP increases locust susceptibility to *M. acridum* [114]. Knockdown of SP inhibitor 1 (Serpin1) markedly decreased *defensin* expression [86].

In addition to AMPs, the PPO system also contributes to the antifungal immune response (Figure 3). The PPO activation system has been basically and similarly elucidated in various insects [115]. PPO is activated by the SP cascade to PO. Then, the PO oxidates Tyrosine or L-DOPA to quinone and ultimately forms melanin. In this process, reactive oxygen (ROX) is produced. ROX and quinone are cytotoxic to pathogens. There are a number of hormones and genes that activate PPO. The eicosanoids and adipokinetic hormone-I (AKH) both stimulate PO activity; however, AKH but not eicosanoids enhance nodules of the body cavity that are induced by laminarin (a short chain β-1,3-glucan) [116,117]. Interestingly, locusts fortified with PO activity by AKH exhibited increased susceptibility to *M. acridum* [118]. The total PO activity of desert locusts gradually decreased with *M. acridum* infection [119]. Except for the PPO activation system, PPO transcription expression is responsive to infection by *Metarhizium* and is regulated by hormones and two genes likewise [87,120]. 20E further promoted the expression of GNBP2 to increase PPO expression in the fat body of locusts infected with *M. anisopliae* [120]. Decreased expression of IAP boosted PO activity during infection by *M. acridum*. Impaired expression of serpin1 reduces PO expression and activity [114].

The Toll pathway has been exhaustively researched in various insects, locusts also have identical pathway and response to fungal infection. In addition, the ecdysone/EcR pathway also regulates the expression of AMPs and other immune genes. Impairment of EcR would weaken the resistance of locusts to *M. anisopliae*. The EcR seems to directly regulate the expression of immune genes by interacting with other transcription regulators. However, there is no evidence showing that EcR could be bound to the transcription factor Dif or Dorsal of the Toll pathway, which regulates the expression of AMPs with antifungal activity. Hence, the connection between ecdysone/EcR and the Toll pathway requires thorough investigation. PPO transcription in the fat bodies is regulated by 20E through GNBP2 under fungal infection. The function of GNBP2 in regulating PPO expression is unclear and it needs to be further studied.

### 3.3. Cellular Response at Late-Phase Immunity

Cellular immunity, including phagocytosis and encapsulation, is mediated by granulocytes, plasmatocytes, and oenocytoids, which differentiate from prohemocytes (Figure 3). The proportion of the different subtypes was also changed by fungal infection. The number of plasmatocytes was reduced, whereas the proportions of granulocytes, spindle-shaped cells, and spherulocytes gradually increased after infection with *M. acridum* [119]. The total hemocyte number fluctuates in locusts with mycosis. The number of total hemocytes was increased first and then decreased compared to that in the mock group after infection with *M. acridum* [90,121]. The total number of hemocytes was slightly lower in infected desert locusts than in the control locusts [119]. IAP maintains hemocyte numbers during infection by *M. acridum* [114]. The partial subtypes of granulocytes and plasmatocytes participate in phagocytosis [90]. These hemocytes directly engulf the hyphal bodies of pathogenic fungi. The phagocyte number first increased and then decreased in infected locusts [90]. The encapsulation is aimed at large invaders such as fungal mycelium. The upregulated expression of the C-type lectin Immunlectin-1 that is mediated by 20E enhances the encapsulation of hemocytes [111].

The differentiation of prohemocytes is induced by *Metarhizium*. The differentiated hemocytes including plasmatocytes, oenocytoids, and granulocytes have diverse functions in defense against fungal infection. A previous study revealed that several genes, including LPS-induced TNF-α transcription factor (LITAF)-like transcription factor (LL3) and genes in the STAT pathway, regulate hemocytes differentiation in the mosquitoes after infection with parasites [122]. In addition, differentiation of hemocytes is regulated by the pathways of JAK-STAT, Notch, Toll, and MAPKs under general physiology [123]. Even so, the regulatory mechanism of hemocyte differentiation in locusts under fungal infection is largely unknown. The receptors and transducers that directly regulate hemocytes differentiation induced by pathogenic fungi are necessary to be found and researched in-depth. Phagocytosis and encapsulation are two main ways to eliminate invading pathogenic fungi. Although there are many studies on other insects, these processes have not been fully discovered yet. Therefore, the differentiation and action of hemocytes needs to be further explored.

## 4. Locust Behavioral Fever and Density-Dependent Disease Resistance

### 4.1. Behavioral Fever

Behavioral fever, an acute change of thermal preference raised by pathogens, has been reported in a variety of invertebrates and ectothermic vertebrates [124]. Locusts that are infected with *M. acridum* initiate behavioral fever [125]. The locust behavioral fever delays the progress of fungal infection [126]. Laminarin, a short chain of β-1,3-glucan, causes fever [126]. The eicosanoid biosynthesis inhibitor, dexamethasone, prevents laminarin-induced fever. This effect is reversed by arachidonic acid [126]. The Toll pathway activates eicosanoid biosynthesis in lepidopteran *Spodoptera exigua* infected by *M. rileyi* [127]. This finding indicates that fungal PAMP stimulates the behavioral fever of the host through an activated immune-related pathway. Destruxin A inhibits locust behavioral fever caused by *M. acridum* [128]. Behavioral thermoregulation cannot change locust feed frequency and food consumption [129]. Thermoregulation can only retard locust death but cannot fully eliminate fungal virulence [129]. Moreover, it does not affect the growth of *M. acridum* [130]. After behavioral fever, gregarious individuals birth more solitarious-phase offspring [14].

Thermoregulation facilitates resistance of locust to mycosis. The behavioral fever of locusts is mediated by the Toll pathway, which triggers the biosynthesis pathway of eicosanoids and consequently activates locusts to regulate body temperature. The regulatory mechanisms, including the behavioral change and the interaction between the Toll pathway and eicosanoid, need to be elucidated.

### 4.2. Density-Dependent Disease Resistance

Density-dependent prophylaxis is proposed by the summarizing the lepidoptera-baculovirus interaction. Virus-induced mortality has declined with increased population density [131]. Gregarious locusts are more resistant to *M. acridum* than solitarious locusts [13]. However, the hatchlings from gregarious locusts have lower resistance against fungal infection than the solitarious locusts [132]. *GNBP3*, *PGRP-SA*, and *attacin* are expressed at higher levels in gregarious locusts than in solitarious locusts [133]. In addition, the cytokine TNF inhibits cellular responses, such as phagocytosis, and elevates humoral responses in the gregarious locusts [134].

## 5. Concluding Remarks

In this review, we revealed that the interactions between *Metarhizium* spp. and locusts the pathogenesis of the fungal pathogen *Metarhizium* to the host locusts, meanwhile the host defense against mycosis by the feat of physical barriers, cellular and humoral immune responses, behavioral fever, and enhanced immunity with increased population density. These main processes are similar in various pathogenic fungi and host insects. The MAPK pathways are important for all processes of fungal infection and for other entomopathogenic fungi, including *Beauveria bassiana*. However, the secreted metabolites and enzymes are rather specific to oppose insect immunity in various fungi. The locusts defense, including humoral and cellular immunity, is basically identical to other insects. The early phase immunity of locust was also found in other insects but it does not respond to fungal infection. In addition, behavioral fever, and density-dependent prophylaxis were also clarified in other insects. However, density-dependent prophylaxis of locusts is different from that of lepidoptera. The phase change caused by increased density in locusts resulted in a remarkable conversion of body physiology. Accordingly, the locust immunity might be unidentified to the lepidoptera.

An intricate and universal model was built to elaborate on the interaction between entomopathogenic fungi and host insects through integration of these studies in *Metarhizium* spp. and locusts. These findings provide sufficient insights into understanding interactions between entomopathogenic fungi and insects. We hope that this review could suggest guidelines for researchers to explore unsolved problems. For example, how the MAPK pathway regulates appressorial formation, how the conversion of cell wall to adapt hemolymph, how early phase immunity responds to fungi on cuticle, and how the prohemocytes differentiate to various hemocytes under fungal infection. The more effectively insecticidal targets and biological agents are developed from the research of interaction for control of agricultural insect pests.

## Data Availability

Not applicable.
